# Comparison of efficacy of shock-wave therapy versus corticosteroids in plantar fasciitis: a meta-analysis of randomized controlled trials

**DOI:** 10.1007/s00402-018-3071-1

**Published:** 2018-11-13

**Authors:** Yuan Xiong, Qipeng Wu, Bobin Mi, Wu Zhou, Yi Liu, Jing Liu, Hang Xue, Liangcong Hu, Adriana C. Panayi, Guohui Liu

**Affiliations:** 1 Department of Orthopedics, Union Hospital, Tongji Medical College, Huazhong University of Science and Technology, Wuhan, 430022 China; 2 Department of Orthopedics, Pu’ai Hospital, Tongji Medical College, Huazhong University of Science and Technology, Wuhan, 430022 China; 3 The division of Plastic Surgery, Brigham and Women’s Hospital, Harvard Medical School, Boston, MA USA

**Keywords:** Plantar fasciitis, Shock-wave, Corticosteroid, Intra-articular

## Abstract

**Background:**

Corticosteroid (CS) injections have been proven to be effective in ameliorating symptoms of plantar fasciitis. Shock-wave (SW) therapy is another common treatment of plantar fasciitis, and several meta-analyses have documented its advantages when compared to placebo treatment. Despite this, few studies have focused on comparing the use of CS and SW in the treatment of plantar fasciitis. The purpose of this meta-analysis is to assess whether SW is superior to CS in managing plantar fasciitis, both in terms of ameliorating pain as well as improving functionality.

**Methods:**

A systematic search of the literature was conducted to identify relevant articles that were published in Pubmed, Medline, Embase, the Cochrane Library, SpringerLink, Clinical Trials.gov and OVID from the databases’ inception to July 2018. All studies comparing the efficacy of SW and CS in terms of pain levels and functionality improvement were included. Data on the two primary outcomes were collected and analyzed using the Review Manager 5.3.

**Results:**

Six studies were included in the current meta-analysis. A significant difference in VAS score (MD = − 0.96, Cl − 1.28 to − 0.63, *P* < 0.00001, *I*^2^ = 96%) was noted between the SW group and the CS group. No significant difference was seen in the Mayo CSS or FFI or HFI or 100 Scoring System score at the 3 months follow-up (Chi^2^ = 0.62, *I*^2^ = 0%, *P* > 0.05).

**Conclusions:**

The clinical relevance of the present study is that both SW and CS were effective and successful in relieving pain and improving self-reported function in the treatment of plantar fasciitis at 3 months. Although inter-group differences were not significant, the VAS score was better improved in the SW group, highlighting that shock-wave therapy may be a better alternative for the management of chronic plantar fasciitis.

## Introduction

Plantar fasciitis is a major cause of heel pain, which is often seen in middle aged and elderly people and is responsible for a reduction in quality of life [1]. Treatment modalities that have been used in plantar fasciitis include orthoses [2, 3], stretching [4, 5], taping [6], extracorporeal shock-wave therapy [7, 8], laser therapy [9], percutaneous injection [10] and drug medication [11]. Corticosteroid (CS) injections have been proven to be effective in achieving beneficial effects for this disorder [12, 13], but there exist limitations. Studies [14, 15] have reported different durations of pain relief due to use of different injection sites, as well as side effects such as heel pad atrophy and rupture of the plantar fascia [15]. Shock-wave (SW) therapy is another common treatment of plantar fasciitis, and several meta-analyses have documented its advantages over placebo treatment [16, 17]. However, the therapeutic response to SW therapy depends on the intensity, pulse cycle and SW modality [16]. Although both methods are useful for plantar fasciitis, few studies have focused on comparing the use of CS and SW in the treatment of the condition and which treatment is superior, if either, is still uncertain. The purpose of this meta-analysis is to assess whether SW is superior in managing plantar fasciitis, both in terms of ameliorating pain as well as improving functionality.

## Materials and methods

### Search strategy

The databases searched were Pubmed, Medline, Embase, the Cochrane Library, SpringerLink, Clinical Trials.gov and OVID from inception to May 2018. The following search terms were used: plantar fasciitis or PF; Shock-wave or SW; Corticosteroid or CS; intra-articular injection or IA injection.

### Data selection

Inclusion eligibility was independently performed by two investigators who screened the title and abstracts of all articles. Disagreements were resolved with discussion between the authors. A third researcher was the adjudicator when the two investigators did not reach agreement. The inclusion criteria were: (1) studies were designed as RCTs; (2) participants were at least 18 years old; (3) studies compared SW with CS; (4) articles were written in the English language.

### Data extraction

Two authors independently extracted the following data from each eligible study: study design, type of study population, age, number of participants and interventions. Any discrepancies in data extraction were resolved by a third investigator.

### Quality and risk of bias assessments

The modified Jadad scale was used to assess the methodological quality of each study. A score of ≥ 4 indicated high quality. The Cochrane Handbook for Reviews of Interventions (Revman Version 5.3) was used to assess the risk of bias. Two independent authors subjectively reviewed all articles and assigned a value of “high”, “low” or “unclear” based on the following items: selection bias; performance bias; detection bias; attrition bias; reporting bias and other bias. Any disagreements were resolved with discussion to reach a consensus. If a consensus could not be reached a third investigator was consulted.

### Statistical analysis

RevMan software was used to analyze the numerical data extracted from the included studies. For binary data, the risk ratios (RR) and 95% confidence intervals (CI) were assessed (*ɑ* = 0.05 for the inspection standards). For continuous data, means and standard deviations (SD) were pooled to a weighted mean difference (WMD) and a 95% confidence internal (CI) in the meta-analysis. Heterogeneity was tested using the I^2^ statistic. Studies with an *I*^2^ statistic of 25–50% were considered to have low heterogeneity, those with an *I*^2^ statistic of 50–75% were considered to have moderate heterogeneity and those with an *I*^2^ statistic > 75% were considered to have high heterogeneity. When the *I*^2^ statistic was > 50%, sensitivity analyses were performed to identify any potential sources of heterogeneity. Statistical significance was indicated by a *p* value < 0.05. And the analysis was done using fixed effects which adds more statistical power to the analysis.

## Results

### Description of studies and demographic characteristics

**Fig. 1:**
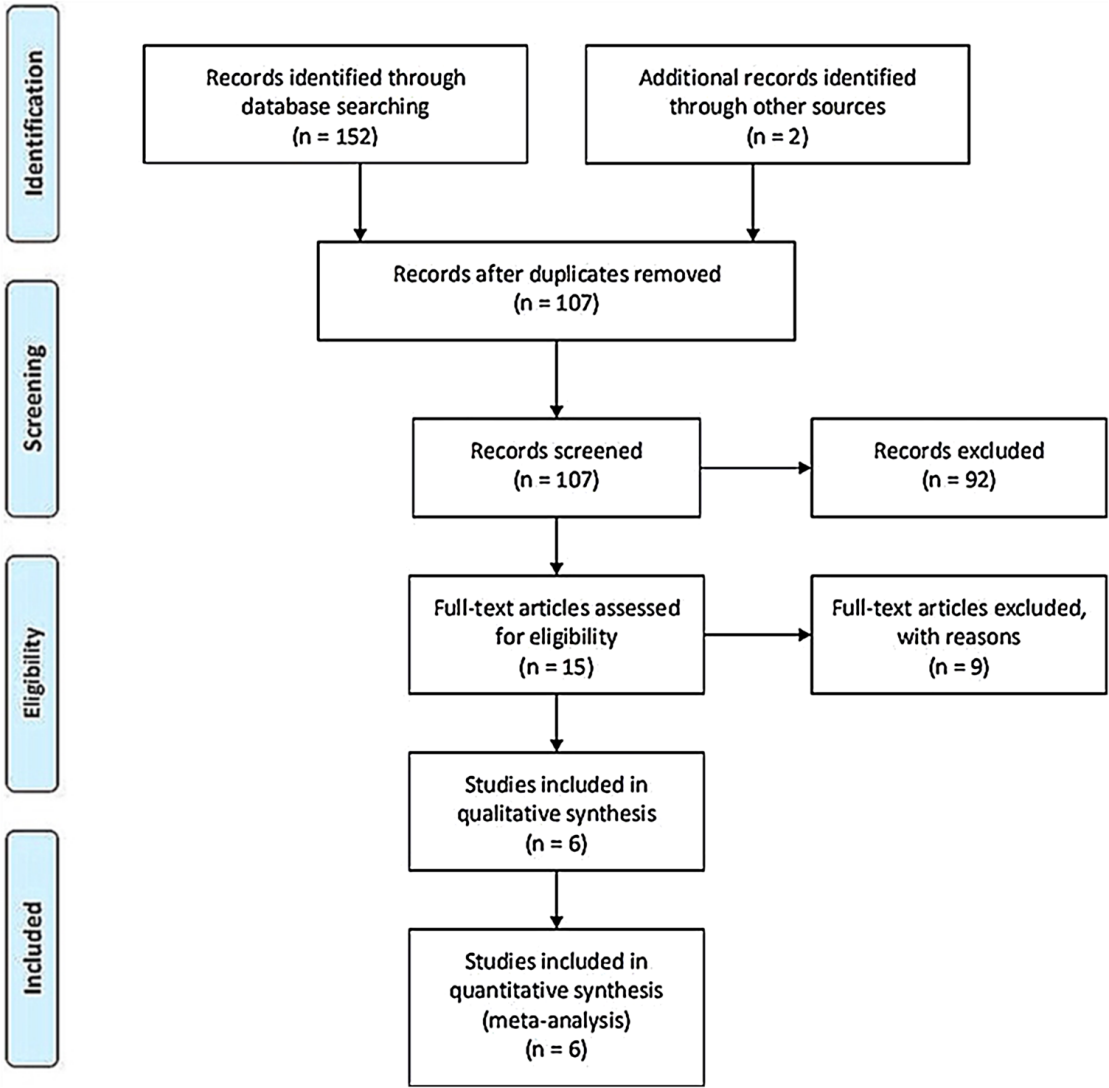
Flow chart outlining the process of study identification, inclusion and exclusion

A total of 154 articles were identified as potentially relevant studies (Fig. 1). Following screening of titles and abstracts (*n* = 92) and removal of duplicates (*n* = 47) resulted in a total of 15 full publications. The 15 full manuscripts were assessed and a further 9 trials were excluded, leaving 6 trials eligible to be included in the meta-analysis. The demographic characteristics are summarized in Tables 1 and 2. All trials compared the effect of the SW group versus the CS group.

**Table 1:** Characteristics of included studies

Study	Year	Country	Patients (*n*)		Age (Y)		Average disease duration, month (week)		PF thickness (mm)		Study design
			SW	CS	SW	CS	SW	CS	SW	CS	
Lai	2018	Taiwan, China	47	50	54.53 ± 8.62	54.58 ± 8.63	7.94 ± 2.92	8.06 ± 2.87	0.37 ± 0.07	0.38 ± 0.06	RCT
Sehriban	2017	Turkey	36	36	50.22 ± 8.29	47.86 ± 7.90	8	9	4.75	4.7	RCT
Mark	2005	Australia	61	64	38.6	39.9	12.7	14.6	NC	NC	RCT
Nayera	2012	Egypt	30	30	34.27 ± 7.19	34.23 ± 6.67	NC	NC	5.94 ± 0.54	5.96 ± 0.46	RCT
Istemi	2010	Turkey	27	33	42.9 ± 7.08	44.7 ± 9.20	37.7 ± 8.6	39.4 ± 10.2	NC	NC	RCT
Fariba	2016	Iran	20	20	41.45 ± 8.05	42.85 ± 8.62	8.5 ± 4.53	10.4 ± 5.53	NC	NC	RCT

**Table 2:** Characteristics of the six trials selected showing general information

Study	Year	Treatment cycle	Treatment schedule		Assessment methods	Adverse event	Follow-up, month
			SW	CS			
Lai	2018	2	The stable energy level 6 (0.29 mJ/mm^2^) was kept for 25 min to achieve total 1500 shock	20 mg triamcinolone acetonite with 2 ml 2% xylocaine were injected into the marker	PFT, VAS, 100-point scoring	NC	3
Sehriban	2017	3	A 15-mm head with 2000 shockwave at each session at 10-Hz frequency with an energy flux density per shock of 0.16 mJ/mm^2^	Single 1-mL dose of betamethasone sodium plus 0.5 mL of prilocaine	VAS, PFT, HTI, FFI	NC	6
Mark	2005	3	3 applications of 1000 pulses of an energy flux density of 0.08/mm^2^	One milliliter betamethasone (5.7 mg) and 2 mL of lignocaine 1% were injected	VAS, TT scoring	NC	12
Nayera	2012	2	Energy intensity applied ranged from 14 to 17 kV, 2 Hz, 1000–1500 pulses, same technique is repeated after two weeks	Injection of 2 mL of 4 mg/mL twice (betamethasone diproprionate and betamethasone sodium phosphate, 0.5% zylocaine hydrochloride)	Mayo CSS PFT	NC	6
Istemi	2010	1	A single application of 3,000 shockwaves Using an electrohydraulic shockwave generator	A 2-mL syringe filled with 0.5 mL of combined betamethasone dipropionate (6.43 mg/mL) andbetamethasone sodium phosphate (2.63 mg/mL)	VAS, HTI	Pain (*n* = 4), no infection	3
Fariba E	2016	5	2000 shockwaves/session of 0.2 mJ/mm^2^, all subjects received 5 sessions of ESWT at 3-day intervals	Injection of 5 ml once a week, 3 times in total	VAS, FFI	NC	2

### Risk of bias in included studies

**Fig. 2:**
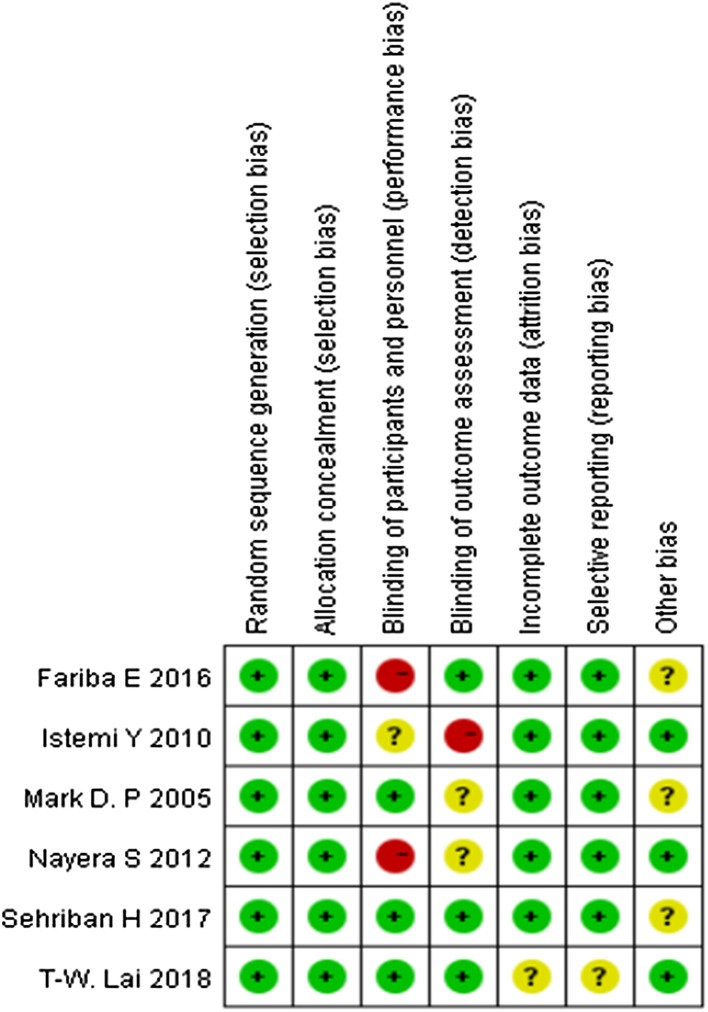
Risk of bias summary

Assessment of the risk of bias is presented in Fig. 2. All trials included were randomized trial designs [18–23]. Three trials [20–22] did not describe the methods of allocation concealment. Blinding of participants and personnel (performance bias) was unclear and incomplete outcome data (attrition bias) was high risk in one trial [20]. One trial [18] had patients lost to follow-up.

### Shock-wave versus corticosteroid

**Fig. 3:**
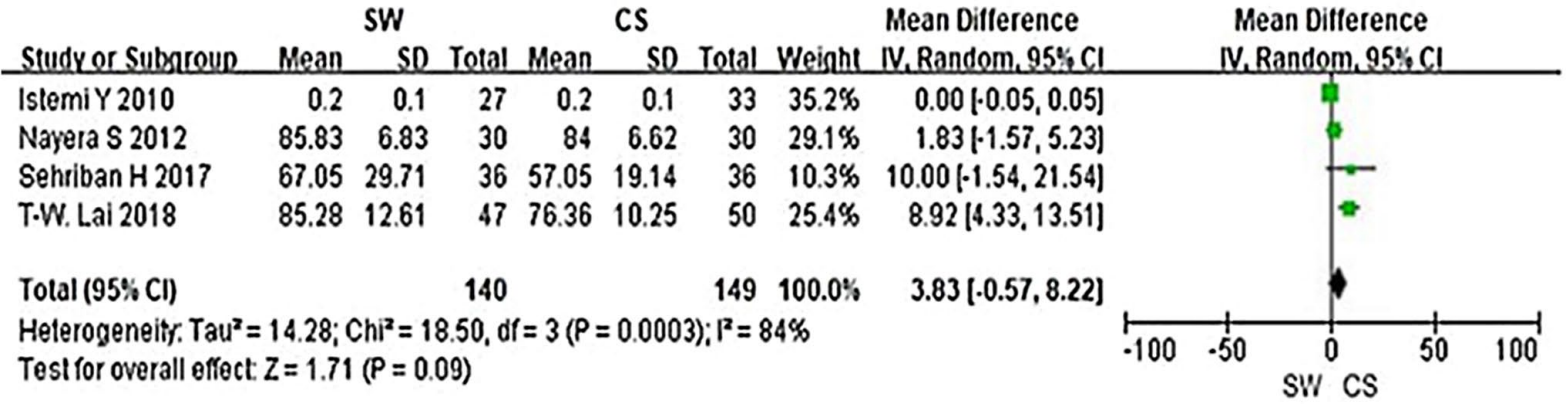
Forest plot of Mayo CSS, FFI, HFI and 100 Scoring System score in the SW group compared with the CS group from the 3-month follow-up

**Fig. 4:**
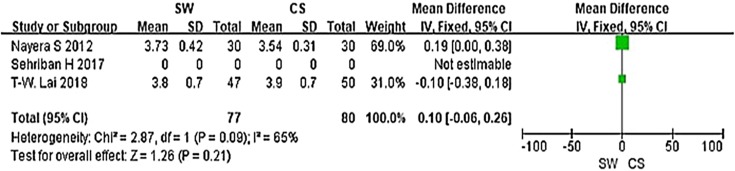
Forest plot of PFT in the SW group compared with the CS group from the 3-month follow-up

**Fig. 5:**
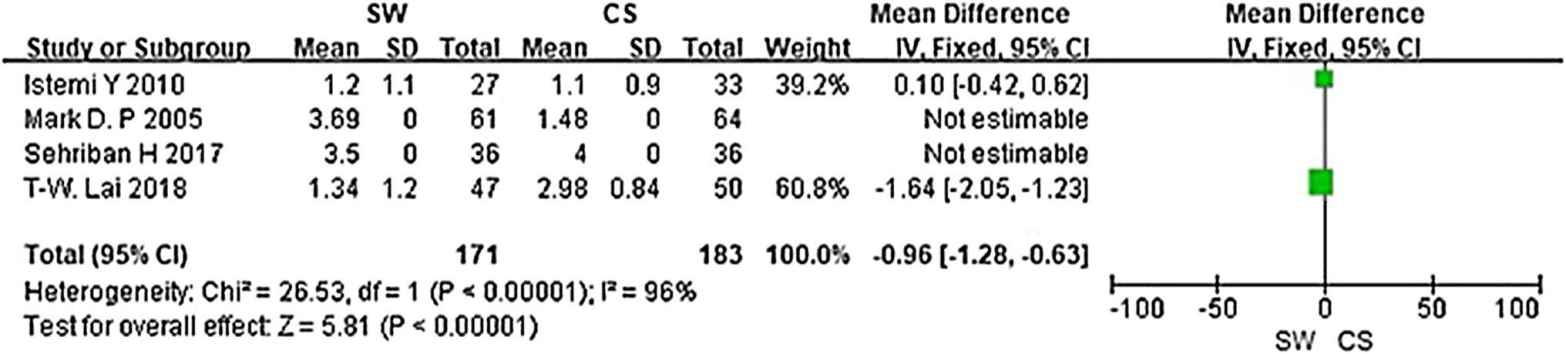
Forest plot of VAS score in the SW group compared with the CS group from the 3-month follow-up

From the six studies comparing SW with CS, four used the Mayo CSS, FFI, HFI and 100 Scoring System score (both including pain and functional subscales) 3 months after accepting treatment. SW was not found to be superior to CS when calculating the pooled effect size of Mayo CSS, FFI, HFI, and 100 Scoring System score from the follow-up at 3 months (Fig. 3; SMD = 3.83, *P* = 0.09, *I*^2^ = 84%). A sensitivity analysis failed to determine any one or two trials that might be causing the statistical heterogeneity. Further analysis of the different changes 3 months after accepting treatment in PFT was performed and is shown in Fig. 4 (MD = 0.10, *P* = 0.21, *I*^2^ = 65%). No significant heterogeneity was observed in this analysis. In addition, trial outcomes assessed by the VAS score were also analyzed, and the result is displayed in Fig. 5 (MD = − 0.96, *P* < 0.00001, *I*^2^ = 96%). A significant difference in VAS score was noted between the SW group and the CS group.

## Discussion

Shock-wave therapy has been used in the treatment of calcified tendonitis of the rotator cuff, nonunion of bone, chronic tennis elbow, and painful heel syndrome [24–27]. The efficacy of SW was controversial in previous studies. The success rates range from 40 to 80% [28–31], and the results are affected by symptom duration [32]. In this meta-analysis, which synthesizes the efficacy of SW on the basis of comparison with CS, SW displayed similar efficacy to CS in improving self-reported function and better effect on relieving pain in the treatment of plantar fasciitis at 3 months.

To our knowledge, this is the first meta-analysis looking at randomized controlled trails comparing the efficacy of SW and CS, and prior to this analysis the outcome of CS and SW as primary treatments of plantar fasciitis remained elusive. In 2012, Saber et al. [21] performed a randomized controlled trial, which showed SW was as useful as CS for relieving symptoms of plantar fasciitis. However, Porter et al. [23] revealed that corticosteroid injection was more efficacious and cost-effective than SW in the treatment of plantar fasciopathy. In our meta-analysis, we found no significant difference in Mayo CSS, FFI, HFI or 100 Scoring System score. A moderately better outcome was, however, seen in the SW group in terms of symptom and pain control in these scoring systems. This difference was not, however, statistically significant (*p* > 0.05). In addition, a significant difference in VAS score was noted between the SW group and the CS group (MD = − 0.96, *P* < 0.00001, *I*^2^ = 96%), which encouraged us to believe that SW is more effective in relieving pain for the treatment of plantar fasciitis.

Previous studies had investigated the relationship of PFT and clinical symptoms. Thickening of the plantar fascia insertion more than 4 mm is considered abnormal and more than 5 mm is suggestive of plantar fasciopathy [33, 34]. As such, it can be inferred that PFT can be treated as an observational index for the comparative research. In our meta-analysis, we observed the different changes in PFT after accepting 3 months treatment and we found no significant difference between the two groups.

Many researchers [35–37] had pointed out that SW was effective in short- and mid-term follow-up in terms of relieving pain and improving functionality, but its efficacy in long-term should be established, especially for the recurrence of plantar fasciitis. Malliaropoulos [36] et al. conducted a retrospective study concerning recurrence rate of plantar fasciitis in 2016, which showed that three key factors for recurrence: female sex, pretreatment pain duration, and the number of SW sessions received. Wang Ching-Jen et al. [37] observed long-term (12 months) results of 79 patients (85 heels) received 1500 impulses of shockwaves at 16 kV to the affected heel in a single session, and the recurrence rate was relative low (11%, 9/81 heels), they also regarded that recalcitrant plantar fasciitis could be caused by plantar fascia thickening and loss of normal tissue elasticity. Therefore if a patient presents with advanced symptoms then they may be less receptive to conservative management. CS as another treatment for plantar fasciitis had some advantages to be recommended, but a Cochrane review concluded that whilst valuable in the short term, the effects of injection therapy are not maintained beyond 6 months [38]. Furthermore, Jolanta et al. [39] had compared the efficacy between ultrasound and shock-wave therapy among 47 patients of plantar fasciitis, which showed that the shock-wave therapy would be a better option for pain relief, as with fewer treatments, the cost of therapy is lower. And the researchers suggested that a complex prophylaxis programme needed be implemented, patients should be encouraged to fight with obesity, and the key to combine theory with practice is to teach patients responsibility and integrate the therapy with everyday life. Thus, we can regard that SW combined with health education would be a better choice for patients compared with ultrasound wave therapy.

Just like other meta-analyses, our study is not devoid of limitations. First, in the SW group, the outcomes may be highly dependent on machine-type (electrohydraulic, electromagnetic, and piezoelectric systems) and treatment protocols [25, 30, 40]. The energy levels are categorized into high (> 0.60 mJ/mm^2^), medium (0.28–0.59 mJ/mm^2^), and low (0.08–0.27 mJ/mm^2^) [41]. In the CS group, outcome may be influenced by the injection dosage, timing, and interval. However, in this meta-analysis, the machine type and energy levels of SW, as well as the injection dosage, timing, and interval were nonuniform among the different trials. Second, the current meta-analysis focuses only on papers published in the English language. Inclusion of studies reported in other languages may influence heterogeneity and affect the current results. In addition, the variance of populations, disease duration, and outcome scores contributed to a high level of heterogeneity and diverse clinical outcomes. In this study, we focus on the shock-wave therapy and corticosteroids therapy for the treatment of Plantar Fasciitis. So we did not look at an untreated control group. All these inconsistencies complicated data synthesis and increased the risk of incorrect results. Furthermore, the follow-up time was not consistent across studies. Further rigorously designed RCTs with larger sample sizes are necessary to better confirm the efficacy of SW.

## Conclusion

The clinical relevance of the present study is that both SW and CS were effective and successful in relieving pain and improving self-reported function in the treatment of plantar fasciitis at 3 months post treatment. Although inter-group differences were not significant, the VAS score showed higher improvement in the SW group, thus shock-wave therapy appears to be a better alternative for the management of chronic plantar fasciitis. Further studies are needed to compare the efficacy of SW and CS on long-term follow-up patients.

## Abbreviations

PFT: Plantar fascia thickness
VAS: Visual Analogue Scale
HTI: Heel tenderness index
TT: Tenderness threshold
FFI: Foot function index
CSS: Clinical scoring system
RCT: Randomized controlled trial
NC: Not clear
